# Culture-sensitive lifestyle intervention tailored to non-Western migrant older adults improves physical performance: A randomized controlled trial

**DOI:** 10.1016/j.jnha.2025.100584

**Published:** 2025-05-22

**Authors:** Esmée J.M. Biersteker, Jantine van den Helder, Nannette van der Spek, Mieke Holwerda, Hinke Kruizenga, Peter J.M. Weijs, Michael Tieland

**Affiliations:** a Department of Nutrition and Dietetics, Faculty of Health, Sport and Physical Activity, Amsterdam University of Applied Sciences, Dokter Meurerlaan 8, Amsterdam, 1067 SM, The Netherlands; b Amsterdam Movement Sciences Research Institute, VU University, Amsterdam, The Netherlands; c Amsterdam Gastroenterology Endocrinology Metabolism Research Institute, VU University, Amsterdam, The Netherlands; d Department of Nutrition and Dietetics, Amsterdam University Medical Centers, VU University, 1081 HV Amsterdam, The Netherlands; e Institute for Physical Activity and Nutrition, School of Exercise and Nutrition Sciences, Deakin University, Geelong, Victoria, Australia

**Keywords:** Sarcopenia, Culture-sensitive, Ethnic minority, Behavioural change, Physical performance, Protein intake

## Abstract

**Objectives:**

To counteract sarcopenia in non-Western migrant older adults, lifestyle interventions with increased physical activity and adequate dietary protein intake are promising. However, regular community-based lifestyle interventions often lack a culture-sensitive approach. The aim of this study was to assess the effects of a newly developed culture-sensitive lifestyle intervention on physical performance in non-Western Surinamese older adults.

**Design:**

A randomized controlled trial.

**Participants:**

This study was conducted with 65 non-Western migrant participants (Surinamese, 65 ± 7 y, 91% female, 82% overweight or obese).

**Intervention:**

The participants were allocated to the culture-sensitive lifestyle intervention (n = 35) or control group (n = 30). The six-month intervention consisted of an exercise training program and a nutritional program, both adapted to the cultural and personal needs of the participants.

**Measurements:**

The primary outcome was physical performance, measured by 6-minute walking test. Secondary outcomes were timed-up-and-go, 30-seconds chair stand, knee-extension strength, single leg stand tests, appendicular lean soft tissue mass, fat mass, protein intake and daily physical activity. Linear mixed models were performed to assess intervention effects with significance set at p < 0.05.

**Results:**

The intervention group improved physical performance by 12% from baseline 440 ± 62 m to 6 months 492 ± 73 m compared to 8% in control group from 438 ± 93 m to 471 ± 66 m (+25.5 m, 95%CI (3.2;47.9), p = 0.027). Knee-extension strength was significantly better maintained in the intervention group from 273 ± 71 N to 270 ± 70 N, whereas the control group decreased knee-extension strength by 8% at six months from 262 ± 78 N to 240 ± 87 N (+19 N, 95%CI (1–38), p = 0.040). The intervention group significantly increased protein intake more (from 63 ± 21 to 78 ± 38 g/day) compared to the control group (from 72 ± 25 to 78 ± 29 g/day) at three months (+15 g/day, 95%CI (1;28), p = 0.035). No difference between groups was found for physical activity or the other secondary outcomes.

**Conclusions:**

In non-Western migrant Surinamese older adults, a culture-sensitive lifestyle intervention improved physical performance, protein intake, and maintained muscle strength, presenting a promising approach to manage sarcopenia risk in this population.

**Trial registration:**

Clinicaltrials.gov (NCT06407583).

## 1 Introduction

The health of non-Western migrant older adults in the Netherlands is poorer compared to the Dutch majority population. In these populations, such as Moroccan, Turkish and Surinamese, the prevalence of multiple chronic diseases like hypertension or type 2 diabetes mellitus is significantly higher and occurs from a younger age [[1], [2], [3]]. A major risk factor for developing chronic diseases is the age-related loss of muscle mass, muscle strength, and muscle function, also referred to as sarcopenia [4]. The prevalence of sarcopenia varies between these migrant groups, with the highest rates observed in South-Asian Surinamese older men (61%) and woman aged 55 years and older (31%) [5].

The development of sarcopenia is influenced by multiple factors, with physical inactivity and insufficient protein intake identified as the most crucial lifestyle related determinants [4,6,7]. Numerous randomized controlled trials have demonstrated the beneficial effects of physical exercise and adequate protein intake for preventing the loss of muscle mass, strength, and function in older adults [[8], [9], [10]]. As such, the World Health Organization (WHO) recommends that older adults should engage in at least 150−300 min a week of moderate physical activity, such as walking, in combination with resistance training twice a week [11]. In addition, daily dietary protein intake of at least 1.0–1.2 g of protein per kilogram bodyweight is advised to preserve muscle mass in older adults [12].

Older adults from non-Western populations often fall short in both guidelines [5,13], due to variations in social, cultural and lifestyle factors which profoundly influence daily life behaviours [14,15]. Health interventions for behavioural change are typically designed for the general Western population and often fail to account for the cultural specific needs of non-Western migrant older adults, underscoring the need for culturally tailored approaches [[16], [17], [18], [19], [20]]. Cultural tailoring in health interventions involves adapting the study design, materials, and intervention components to align with the social and cultural norms, beliefs, and preferences of the target population [18,21]. For instance, dietary habits, including protein intake, are deeply influenced by cultural and religious practices, which can shape the types of foods consumed, timing of meals, and their social context [13,22]. Existing interventions often overlook these nuances, leading to a mismatch between recommendations and what is feasible or acceptable within these communities. Recognizing and integrating these factors is crucial to improving engagement, participation and subsequently improve healthy lifestyle behaviours.

To optimize protein intake and exercise behaviours in non-Western migrant populations, a culturally tailored lifestyle intervention is essential. Consequently, a new intervention was developed using the intervention mapping methodology, in collaboration with key stakeholders and end-users, including older adults from the target population and healthcare providers. This study aims to assess the effects of this culture-sensitive lifestyle intervention on physical performance in non-Western Surinamese older adults.

## 2 Materials and methods

### 2.1 Study design

**Fig. 1:**
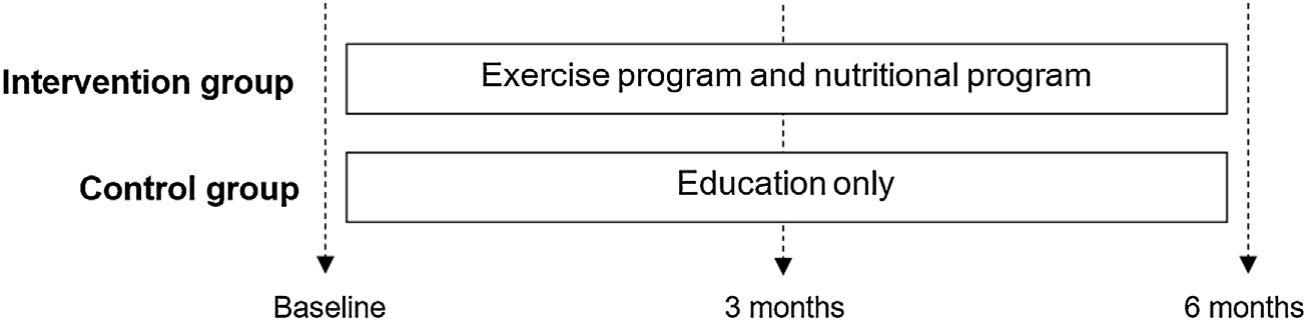
Study design illustrating the time points of measurements.

A single-blind randomized controlled trial with two parallel arms was used to test the effects of this culture-sensitive lifestyle intervention. The lifestyle intervention included an exercise program focused on increasing physical performance and a nutritional program focused on increasing protein intake. Measurements were collected at baseline, after three and six months (Fig. 1). The study ProMIO2.0 was approved by the Medical Ethics Committee (METC) of the Amsterdam University Medical Centers, location VUmc, The Netherlands (NL75885.029.21), and registered at Clinicaltrials.gov (NCT06407583). This study report followed the CONSORT guidelines for reporting randomized trials (see Appendix A). All data related to measurements and intervention were managed using Castor EDC system (Amsterdam, The Netherlands).

### 2.2 Participants

Participants were recruited in Amsterdam, The Netherlands, between May and November 2022 through the distribution of flyers and the local networks of healthcare professionals and general practitioners. We were able to recruit Surinamese older adults (e.g. Creole, Hindustani and other) in this study. Inclusion criteria required that participants must be of non-Western migrant backgrounds, aged 55 years or older, physically capable and willing to participate in the intervention, and able to provide written informed consent. Exclusion was based on the presence of specific medical diagnoses, such as renal insufficiency (Modification of Diet in Renal Disease < 30) [23], unstable coronary heart disease, decompensated heart failure, uncontrolled arrhythmias, chronic obstructive pulmonary disease, advanced stage cancer (phase IV) and degenerative neurocognitive disorders. After approval of the study physician, participants were randomly allocated (1:1) to either the intervention or control group by an independent researcher using computer-generated randomization. The allocation was concealed from the participants, the healthcare professionals and the study assistant until the start of the intervention. The researcher (EB) was not involved (blinded) in multiple aspects of the study, including randomization, screening, assessments, or the execution of the intervention. Additionally, those responsible for the intervention were not blinded, and those responsible for the assessments were blinded to the allocation.

### 2.3 Lifestyle intervention

The lifestyle intervention, delivered by local healthcare professionals and group fitness trainers, aimed to optimize exercise and dietary habits based on health guidelines of the WHO and recommendations on dietary protein intake [11,12]. Participants in the lifestyle intervention group received a personalized exercise program and subsequently, a nutritional program. Similar to other interventions, professional support was gradually reduced over the six-month period to promote self-regulatory behaviour. The high professional support phase of the first three months is characterized by frequent contact with a professional. The subsequent moderate professional support phase of three months is characterized by less contact with a professional to encourage the development of self-regulatory and sustainable behaviour. Participants in the control group only received an informative group session on healthy lifestyles, focusing on exercise and nutritional habits in the first month. After completion of the study, the control group was offered the same information and materials, as the intervention group received during the study. Cost-effectiveness questionnaires confirmed that control participants did not engage in other programs during the study. The key components of the lifestyle intervention are outlined below, with additional details on the developed programs and culture-sensitive aspects available in Appendix B, which includes a figure illustrating the structure. Adherence to the consultations, informative sessions and group training during the intervention was monitored by the physical therapist, group exercise trainer and dietitian.

#### 2.3.1 Exercise program

Participants received the exercise program to enhance physical performance. The goal of the exercise program was reaching the WHO physical activity guidelines for older adults, which recommend 150−300 min of moderate-intensity aerobic activity per week, with emphasize on walking, and incorporating resistance, balance, and functional exercises twice weekly [11]. During the first month, participants trained under supervision of a group fitness trainer in small groups (approximately 6–8 participants) twice a week and were advised to walk at least 150 min per week. The group training took place in our research centre located in their neighbourhood, ensuring proximity to participants’ homes. Each group training session consisted of a 5-min warm up, balance exercises, progressive resistance exercises (e.g. squats, push-ups, chair rises, and vertical rowing using free weights or elastic bands) and a cooling down with flexibility exercises. Each exercise consisted of three sets: the first two sets targeted twelve repetitions maximum, while the third set was performed until failure. If participants exceeded 15 repetitions in the third set, the exercise load was increased, or the exercise variation was made more challenging. Training efforts were recorded for each participant during every session to monitor progress. In the second and third month, one group training was replaced by a home-based training with a personalized program. The home exercises closely matched those from the group training sessions, ensuring they were safe, accessible, and familiar. Participants were provided with elastic bands and small dumbbells (low weighs) and had access to instructional videos demonstrating the home-based exercises. The participants continued the last three months with group training once in two weeks and were advised to perform twice weekly home-based training. The recommended walking duration was increased to 150−300 min per week. Two informative group sessions and individual consultations with a physical therapist were provided to enhance participants' knowledge, awareness, and skills for successfully completing the program. These sessions aimed to motivate participants to follow their exercises at home and achieve a goal of at least 150 min of walking per week. The physical therapist also personalized the program, tailoring it to each participant's physical abilities and limitations while encouraging them to meet their walking targets.

#### 2.3.2 Nutritional program

The nutritional program focused on achieving a daily protein intake of 1.2 g per kilogram of bodyweight per day [12]. If participants were overweight (BMI above 27.5) the adjusted bodyweight in kilograms of a BMI of 27.5 was used. A dietitian implemented the dietary protein intervention, which included three informative group sessions and individual consultations, overall aiming for a minimum daily intake of 1.2 g per kilogram of bodyweight [24] and were guided to achieve a protein intake of >25 g per meal. Participants received recommendations emphasizing the importance of dietary protein, optimal protein intake per meal, and cultural and traditional sources of protein in regular food products. At the beginning of the program, the participants received the culture-sensitive materials that were developed for this program. The examples of a protein poster and pocket booklet are present in Appendix B.

### 2.4 Design culture-sensitive lifestyle intervention

This culture-sensitive lifestyle intervention was specifically developed to address the socio-cultural needs of non-Western migrant older adults, aiming to enhance their engagement and identification with both the exercise and nutritional programs. Based on our previous studies and methods (such as focus groups), we codesigned the following aspects of the intervention considering social norms and values: 1) group-based training sessions as core format for the exercise program, 2) walking as preferred physical activity, 3) focus on raising knowledge and awareness about protein-rich foods and exercise, and their importance for healthy aging [13], and 4) practical guidance on integrating adequate protein intake into daily routines, considering cultural aspects and food product preferences [22]. In addition to the tailored modulation of the intervention, culture-sensitive elements were primarily incorporated into the intervention materials and reinforced through the training and peer consultation of healthcare professionals.

First, the intervention materials were specifically designed to include culturally tailored educational content, condensed messages supported by visual aids, and culturally specific recipes. Second, all healthcare professionals involved in the intervention underwent comprehensive training and monthly peer consultation sessions. This training was designed to equip them with the knowledge and skills necessary to address the specific needs and challenges of engaging with older non-Western migrant adults. A comprehensive overview of the design of the lifestyle intervention is presented in Appendix B.

### 2.5 Measurements

Participant characteristics such as age, sex, body mass index (BMI kg/m^2^), ethnicity, living situation, highest educational level, lifestyle behaviours and medical history were collected at baseline. The primary and secondary outcomes measures were assessed at baseline, at three months and at six months.

#### 2.5.1 Primary outcome

The primary outcome was physical performance measured with the six-minute walk test [[25], [26], [27], [28]]. The participants walked rounds of twenty meters and were asked to cover the maximum distance achievable within the six-minute time frame.

#### 2.5.2 Other physical performance outcomes

Additional outcomes for physical performance were the timed up and go test, 30-seconds chair stand test, knee-extension strength and open and closed eye single leg stance test. The timed up and go test was assessed with a 45−47 cm chair. Participants were instructed to stand up from the chair, walk around a cone positioned three meters in front of the chair and return to a seated position as fast as possible [29]. Time was recorded in seconds and the fastest of three trials was used in analysis. The 30-seconds chair stand test measured the number of times participants could stand up from a seated position within 30 s, keeping their arms crossed in front of their chest [30]. Knee-extension strength was measured in Newton (N) using a hand-held dynamometer (MicroFET) as participants gradually increased their knee extension force to a maximum effect while seated with their knees at a 90° angle. The procedure was repeated twice for each leg and the highest score from the dominant leg was used for analysis [31]. The single leg stand test measured the time in seconds that participants could stand on one leg with eyes open and closed, while keeping their arms on the hips [32].

#### 2.5.3 Body composition outcomes

Body composition was measured using multiple outcomes. Body weight was reported to the nearest 0.1 kg using a calibrated weighing scale (Seca) and height was reported to the nearest 0.01 m using a stadiometer. Appendicular lean soft tissue mass and fat mass were measured using the Bodystat 500 Bioelectrical Impedance Analyses [33]. Appendicular lean soft tissue mass was estimated using the formula of Sergi et al. (2016), which is suitable for older adults and incorporates bodyweight: −3.964 + (0.227 × (Length^2^/Resistance)) + (0.095 × Bodyweight) + (1.384 × Sex) + (0.064 × Reactance) [34]. Muscle quantity of the rectus femoris and vastus lateralis was assessed using 2D ultrasound echography with the Philips Lumify L12-4 linear array transducer. Imaging was performed at the midpoint of the right thigh with a consistent and minimal pressure to avoid compression of the muscles [35]. Subsequently, the acquired images were processed into a comprehensive transverse image, and muscle quantity was determined by measuring the cross-sectional area of the rectus femoris and vastus lateralis.

#### 2.5.4 Behavioural outcomes

Dietary protein intake was derived from a 48-h recall [36]. Participants were asked to retrospectively report their total food intake over two days during a private session with a dietitian in training, conducted during the measurement visit. This approach was implemented to account for participants with low literacy levels and in order to have complete and accurate daily data. Records were coded afterwards with the Dutch Food Composition database (Dutch NEVO Database, version 23) and analysed with the Amsterdam University of Applied Sciences (AUAS) developed syntax using IBM SPSS Statistics version 29.

Daily physical activity was monitored using a three-dimensional Physical Activity Monitor (PAM AM400, Atris BV, The Netherlands), which participants wore around their ankle for seven consecutive days. The recorded movements were categorized into daily minutes of light, moderate, and heavy physical activity. For the analysis, the mean minutes of each activity intensity level were calculated, divided by the total number of complete days the PAM physical activity monitor was worn. In cases where participants wore the monitor for only one full day, only that day’s data were included in the analysis.

Adherence to the consultations, informative sessions and group training during the intervention was monitored by the physical therapist, group exercise trainer and dietitian. In addition, the relevance of the six-minute walk test was examined by categorize improvements into meaningful change. Based on Perera et al. (2006), a small meaningful change was defined if improvements exceeded 20 m or more [37]. The percentage of participants meeting this improvement was reported.

### 2.6 Statistical analysis

Sample size was calculated with a mean difference in change in distance covered during the 6-minute walk test of 24 m and a standard deviation (SD) of 47 m in the intervention group [38]. Using a significance level (α) of 0.05, a power of 80%, and an expected drop-out rate of 30%, a sample size of 63 older adults per group was assumed to be sufficient to detect a statistically significant difference in physical performance. The initial sample size calculation included 126 older adults across three ethnic groups: Turkish, Moroccan, and Surinamese. However, post-pandemic, we encountered considerable difficulties in engaging the Turkish and Moroccan communities for research participation. In contrast, recruitment among the Surinamese population remained more accessible and effective, as individuals from this population showed willingness to participate in research. Consequently, we chose to focus exclusively on the Surinamese group and increased the sample size for this population accordingly.

Data was analysed by the intention-to-treat and per protocol principle and according to a predefined statistical analysis plan. Baseline characteristics were presented as means ± SD for continuous variables when data is normally distributed whereas as medians (25th–75th percentiles) when skewed, and as frequencies and proportion for categorical variables. Baseline differences between the intervention and control group were analysed using the independent sample *t*-test or Mann-Whitney *U* test for continuous data, and X^2^ test or Fishers exact test for categorical data. Baseline and follow-up data for outcome measurements were expressed as mean with SD. Linear mixed models analysis was performed to analyse changes between the intervention group compared to the control group after three and six months. Time and the interaction between time and intervention were added as fixed factors and subjects as random intercept [39]. Covariates such as age, sex, BMI, were added to adjust for confounding effects. Intervention effects were reported as difference with 95% confidence interval and p-values. A p-value of 0.05 was considered to be statistically significant. The statistical analysis plan and data of this study is available in a data package on the Figshare repository with restricted access in consultation with open science principles (https://doi.org/10.21943/auas.28016189).

## 3 Results

**Fig. 2:**
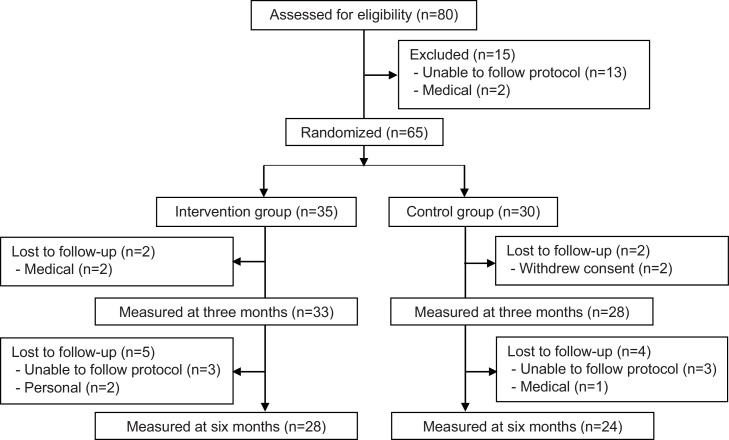
CONSORT flow diagram of participants.

In total, 65 participants were randomized into the intervention group (n = 35) and control group (n = 30). In total, drop-outs were due to the inability to comply with the protocol or personal reasons (n = 8; e.g. long holidays/family obligations), medical (n = 3), and withdraw without reason (n = 2) (Fig. 2). The included participants had a mean age of 65 years (SD = 7), were predominantly female (91%), 82% were classified as overweight or obese, and 66% presented with multimorbidity (2 or more diseases), including 22% with diabetes and 58% with one or more cardiovascular problems. Baseline characteristics did not significantly differ between the intervention and control group (Table 1).

**Table 1:** Participants characteristics summarized by mean ± SD for continuous variables and n (%) for categorical variables.

	Intervention group (n = 35)	Control group (n = 30)
Age	66 ± 7	65 ± 7
Female, n (%)	31 (89)	28 (93)
Bodyweight (kg)	77 ± 13	77 ± 16
Height (cm)	162 ± 7	160 ± 8
BMI (kg/m^2^)	29 ± 4	30 ± 6
Normal weight, n (%)	6 (17)	6 (20)
Overweight, n (%)	13 (37)	11 (37)
Obese, n (%)	16 (46)	13 (43)
Ethnicity, n (%)		
Creole	17 (49)	15 (50)
Hindustani	14 (40)	10 (33)
Other	4 (11)	5 (17)
Living situation, n (%)		
Alone	21 (60)	20 (67)
Together	14 (40)	10 (33)
Level of education, n (%) ƚ		
Low	7 (20)	7 (23)
Intermediate	12 (34)	10 (33)
High	16 (46)	13 (43)
Current smoking, n (%)	4 (11)	1 (3)
Current alcohol consumption, n (%)	18 (51)	14 (47)
Morbidities, n (%)		
Diabetes	8 (23)	6 (20)
Hypertension	19 (54)	10 (33)
Cholesterol	13 (37)	10 (33)
Cardiovascular	10 (29)	4 (13)
Asthma/COPD	3 (9)	3 (10)
Arthrosis	8 (23)	7 (23)
Musculoskeletal and connective tissue disorders	14 (40)	13 (43)
Other	13 (37)	10 (33)

SD, standard deviation; BMI, body mass index.

Current smoking: below 5 cigarettes per day.

Current alcohol consumption: below or even as 7 glasses on average per week.

ƚ Education: Highest obtained educational degree. Low level of education: secondary education, intermediate level: secondary vocational education (MBO), high level: higher vocational education or university (HBO).

### 3.1 Primary and secondary outcomes

**Fig. 3:**
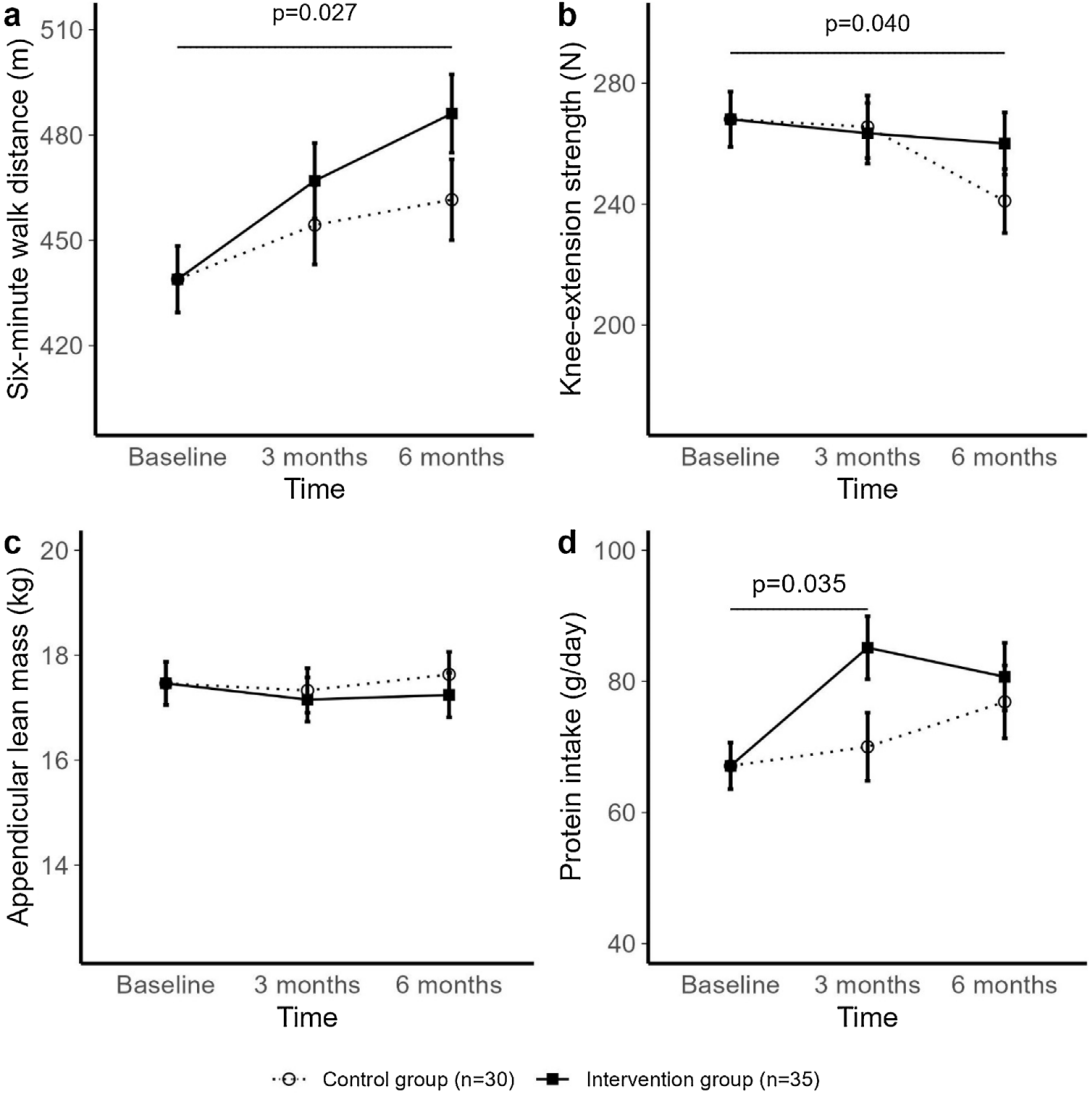
Intervention effects on physical performance(a), knee-extension strength(b), appendicular lean soft tissue mass(c) and protein intake(d). Outcomes are reported as estimated marginal means with standard error bars.

Physical performance measured by 6-minute walking test significantly increased by 12%, from 440 ± 62 m to 492 ± 73 m at six months in the intervention group, compared to 8% in the control group from 438 ± 93 m to 471 ± 66 m (Table 2 and Fig. 3: +25.5 m, 95%CI (3.2–47.9), p = 0.027). In addition, knee-extension strength was significantly maintained in the intervention group from 273 ± 71 N to 270 ± 70 N, whereas in the control group knee-extension strength decreased by 8% at six months, from 262 ± 78 N to 240 ± 87 N (Table 2: +19 N, 95%CI (1–38), p = 0.040). Moreover, closed eyes single leg stand significantly decreased in the intervention group at six months from 4.4 ± 2.6 s to 3.4 ± 2.1 s, and increased in the control group from 4.2 ± 2.8 s to 6.5 ± 11.8 s (Table 2: −3 s, 95%CI (−6 to −1), p = 0.035). However, this result should be interpreted with caution, as its reliability may be compromised due to data skewness and suboptimal assessment conditions. No differences between groups were found for the secondary physical performance outcomes timed up and go and 30-seconds chair stand test, nor were differences found for the body composition outcomes such as appendicular lean soft tissue mass, fat mass and the cross-sectional area of the rectus femoris and vastus lateralis (Table 2).

**Table 2:** Intention-to-treat effects of the lifestyle intervention on physical performance and muscle mass outcomes.

						Linear mixed models with interaction effects *		Adjusted linear mixed models with interaction effects ƚ	
		Intervention group		Control group		Intervention effects		Intervention effects	
Outcome variable		n	Mean ± SD	n	Mean ± SD	Difference (95% CI)	P-value	Difference (95% CI)	P-value
6-minute walk test (m)	0 months	35	440 ± 62	30	438 ± 93				
	3 months	33	474 ± 66	27	455 ± 87	+12.6 (−8.5; 33.7)	0.245	+13.5 (−7.4; 34.9)	0.209
	6 months	27	492 ± 73	24	471 ± 66	+24.5 (2.1; 47.0)	**0.035**	+25.5 (3.2; 47.9)	**0.027**
Timed up and go (s)	0 months	34	8.0 ± 1.8	30	8.0 ± 1.8				
	3 months	33	7.3 ± 1.4	28	7.3 ± 1.3	+0.1 (−0.4; 0.6)	0.586	+0.1 (−0.4; 0.6)	0.626
	6 months	28	7.2 ± 1.3	24	7.3 ± 1.4	+0.1 (−0.4; 0.6)	0.740	+0.1 (−0.5; 0.6)	0.813
30-seconds chair stand test	0 months	34	10 ± 3	30	11 ± 3				
	3 months	34	12 ± 3	28	11 ± 2	+0.4 (−0.9; 1.6)	0.571	+0.4 (−0.8; 1.7)	0.520
	6 months	28	12 ± 2	24	11 ± 2	+0.6 (−0.8; 1.9)	0.430	+0.6 (−0.8; 2.0)	0.381
Knee-extension strength (N)	0 months	35	273 ± 71	30	262 ± 78				
	3 months	33	269 ± 62	28	263 ± 82	−2 (−19; 15)	0.806	−2 (−19; 15)	0.825
	6 months	28	270 ± 70	24	240 ± 87	+19 (1; 37)	**0.043**	+19 (1; 38)	**0.040**
Open eyes single leg stand (s)^a^	0 months	35	26 (14–55)	30	31 (15–78)				
	3 months	33	30 (12–77)	28	26 (10–77)	+5 (−12; 21)	0.598	+4 (−13; 20)	0.658
	6 months	28	44 (12–70)	24	35 (10–101)	−3 (−22; 15)	0.714	−4 (−21; 14)	0.693
Closed eyes single leg stand (s)^a^	0 months	35	3.7 (2.8–5.5)	30	3.6 (2.3–5.6)				
	3 months	33	2.0 (1.5–3.5)	28	2.9 (1.6–5.4)	−2 (−5; 1)	0.116	−2 (−5; 1)	0.088
	6 months	28	3.2 (1.8–4.1)	24	3.6 (1.8–6.1)	−3 (−6; −1)	**0.040**	−3 (−6; −1)	**0.035**
Body weight (kg)	0 months	35	76.7 ± 13.0	30	77.2 ± 16.4				
	3 months	34	76.1 ± 13.1	28	75.6 ± 16.7	−0.1 (−1.1; 1.0)	0.896	−0.1 (−1.1; 1.0)	0.955
	6 months	28	75.1 ± 13.6	24	77.2 ± 18.1	−0.7 (−1.9; 0.4)	0.224	−0.7 (−1.8; 0.5)	0.242
Appendicular lean soft tissue mass (kg)^1^	0 months	33	18.0 ± 3.2	30	16.9 ± 3.5				
	3 months	33	17.6 ± 2.8	28	16.6 ± 3.6	−0.2 (−0.6; 0.2)	0.379	−0.1 (−0.5; 0.3)	0.501
	6 months	27	17.5 ± 2.6	24	17.2 ± 4.0	−0.4 (−0.8; 0.1)	0.066	−0.4 (−0.8; 0.1)	0.096
Fat mass (kg)	0 months	33	33.0 ± 7.8	30	35.1 ± 11.0				
	3 months	33	33.2 ± 7.8	28	34.5 ± 11.1	−0.1 (−0.8; 0.8)	0.992	−0.1 (−1.0; 0.7)	0.738
	6 months	27	32.7 ± 8.0	24	34.5 ± 12.1	−0.1 (−0.9; 0.9)	0.972	−0.2 (−1.0; 0.7)	0.725
Cross-sectional area Rectus Femoris (cm^2^)	0 months	34	4.7 ± 1.4	30	4.9 ± 1.6				
	3 months	33	4.5 ± 1.6	26	4.5 ± 2.1	+0.2 (−0.4; 0.8)	0.566	+0.2 (−0.4; 0.8)	0.522
	6 months	28	4.7 ± 1.5	24	4.9 ± 2.2	+0.1 (−0.5; 0.7)	0.805	+0.1 (−0.5; 0.7)	0.757
Cross-sectional area Vastus Lateralis (cm^2^)	0 months	33	16.5 ± 3.1	29	15.6 ± 5.0				
	3 months	33	15.8 ± 3.4	25	15.7 ± 4.9	−0.4 (−2.0; 1.3)	0.666	−0.2 (−1.9; 1.4)	0.772
	6 months	28	16.2 ± 3.4	24	15.8 ± 5.7	+0.1 (−1.6; 1.8)	0.936	+0.2 (−1.5; 1.9)	0.795

Data are presented as mean ± SD for each time point. Data were analysed using Linear Mixed Models with time and time x intervention interaction as fixed effects and subjects were added as a random intercept. Control group and baseline values are used as reference.

1 Appendicular lean soft tissue mass was assessed by BIA and estimated using the formula of Sergi et al. (2016).

* Crude β-coefficients and 95% CIs are shown for the time x intervention interaction.

ƚ Adjusted β-coefficients and 95% CIs are shown for the time x intervention interaction. Adjusted for age, sex and BMI.

a Data for this outcome is presented as median with interquartile range.

Dietary protein intake data are presented in Table 3 and Fig. 3 as g/day and g/adjusted kg bodyweight/day. Dietary protein intake significantly increased in the intervention group (from 63 ± 21 to 78 ± 38 g/day) compared to the control group (from 72 ± 25 to 78 ± 29 g/day) at three months (+15 g/day, 95%CI (1–28), p = 0.035). Protein intake per adjusted bodyweight increased in the intervention group from 0.9 ± 0.3 to 1.2 ± 0.5 g/kg/day at three months (+0.2 g/kg/day, 95%CI (0.0–0.4), p = 0.052) and to 1.1 ± 0.5 g/kg/day at six months. Baseline protein intake of the control group was 1.1 ± 0.4 g/kg/day and did not change over time. At baseline, the intervention group engaged in 68 m of moderate physical activity per day, compared to 54 m in the control group. The intervention did not significantly change light, moderate, vigorous, or total physical activity between the intervention and control groups (Table 3).

**Table 3:** Intention-to-treat effects of the lifestyle intervention on behavioural outcomes.

						Linear mixed models with interaction effects *		Adjusted linear mixed models with interaction effects ƚ	
		Intervention group		Control group		Intervention effects		Intervention effects	
Outcome variable		n	Mean ± SD	n	Mean ± SD	Difference (95% CI)	P-value	Difference (95% CI)	P-value
Protein intake (g/day)	0 months	35	63 ± 21	30	72 ± 25				
	3 months	33	83 ± 33	28	71 ± 23	+15 (2; 29)	**0.029**	+15 (1; 28)	**0.035**
	6 months	28	78 ± 38	24	78 ± 29	+4 (−11; 18)	0.604	+3 (−11; 18)	0.666
Protein intake (g/adjusted kg/day)	0 months	35	0.9 ± 0.3	30	1.1 ± 0.4				
	3 months	33	1.2 ± 0.5	28	1.1 ± 0.3	+0.2 (0.1; 0.4)	**0.047**	+0.2 (0.0; 0.4)	0.052
	6 months	28	1.1 ± 0.5	24	1.2 ± 0.5	+0.1 (−0.2; 0.2)	0.816	+0.1 (−0.2; 0.2)	0.913
Light physical activity (minutes)	0 months	28	120 ± 45	25	94 ± 38				
	3 months	30	106 ± 37	26	87 ± 24	+3 (−13; 18)	0.728	+3 (−13; 18)	0.733
	6 months	25	107 ± 51	21	101 ± 35	−6 (−22; 11)	0.509	−6 (−23; 11)	0.503
Moderate physical activity (minutes)	0 months	28	68 ± 28	25	54 ± 27				
	3 months	30	58 ± 26	26	54 ± 27	−1 (−13; 12)	0.946	−1 (−13; 13)	0.966
	6 months	25	63 ± 30	21	54 ± 21	+6 (−9; 20)	0.448	+5 (−9; 20)	0.459
Heavy physical activity (minutes)	0 months	27	4 ± 4	23	5 ± 5				
	3 months	30	4 ± 6	26	4 ± 4	+1 (−2; 2)	0.773	+1 (−2; 2)	0.762
	6 months	25	4 ± 3	21	4 ± 3	+1 (−2; 3)	0.713	+1 (−2; 3)	0.712
Total physical activity (minutes)	0 months	28	192 ± 70	25	153 ± 62				
	3 months	30	168 ± 58	26	145 ± 51	+2 (−25; 29)	0.877	+2 (−25; 30)	0.859
	6 months	25	175 ± 77	21	159 ± 53	−1 (−30; 29)	0.987	−1 (−30; 29)	0.983

Protein intake was corrected for BMI > 27.5.

Data are presented as mean ± SD for each time point. Data were analysed using Linear Mixed Models with time and time x intervention interaction as fixed effects and subjects were added as a random intercept. Control group and baseline values are used as reference.

* Crude β-coefficients and 95% CIs are shown for the time x intervention interaction.

ƚ Adjusted β-coefficients and 95% CIs are shown for the time x intervention interaction. Adjusted for age, sex and BMI. Protein intake (g/adjusted kg/day) was adjusted for age and sex.

### 3.2 Adherence to the culture-sensitive lifestyle intervention

This new culture-sensitive lifestyle intervention demonstrated high participant compliance. The average attendance rate for group training sessions was 79% during the high professional support phase and 77% during the moderate professional support phase. Informative group sessions had an average attendance rate of 77%. Attendance rates for dietitian and physical therapist consultations were 91% and 93%, respectively. The adherence of ≥80% to group training sessions was achieved by 54% of the participants in the intervention group. Subsequent per protocol analysis showed similar results to the intention-to-treat analysis (Appendix C; Tables 4 and 5). Among participants in the intention-to-treat dataset, 54% achieved a small meaningful improvement of at least 20 m in the six-minute walk test, compared to 74% in the per protocol dataset.

## 4 Discussion

This randomized controlled trial demonstrated that a six-month culture-sensitive lifestyle intervention improved physical performance, strength and daily protein intake in non-Western Surinamese older adults. We found no effect on body composition and daily physical activity.

Our study demonstrated a significant improvement in physical performance, as assessed by the 6-minute walking test compared to the control group, with walking distance increasing by 12%. While this represents a small change, it is a meaningful improvement of on average 25.5 m for participants in the intervention group and 32.9 m for participants who adhered to the intervention [37]. Our findings align with previous studies, including a study that found a six-month behavioural walking intervention enhanced 6-minute walking distance in older adults with peripheral artery disease [40]. Furthermore, a randomized controlled trial conducted in long-term care settings revealed that residents participating in an exercise program achieved greater improvements in the 6-minute walking test compared to those engaged in a walking-only intervention [41]. Our improvement in physical performance further highlights the potential benefits of an exercise program combined with walking advice as an effective strategy for older adults. Even modest gains in walking distance are associated with higher independence and participants’ quality of life by enabling them to perform daily activities with greater ease and confidence, ultimately supporting healthier aging [42,43].

The intervention group maintained muscle strength in our study, while the control group experienced a 8% decline in muscle strength over six months. This decline aligns with the well-documented consequence of age-related sarcopenia, which progressively reduces muscle mass and function in older adults [4,44]. The observed decline in the control group underscores the importance of targeted interventions to mitigate these effects. While our multicomponent intervention effectively preserved muscle strength, it is important to note that structured resistance exercise training has been shown to elicit more pronounced strength gains. Previous studies have reported strength increases exceeding 30% following three months of resistance exercise training in older adults [10,45]. These findings suggest that although our intervention successfully prevented muscle loss, more intensive resistance training protocols may be necessary to achieve substantial strength improvements. Nonetheless, the maintenance of muscle strength observed in our intervention group highlights the effect of a comprehensive approach that integrated balance, resistance, and functional exercises with a structured nutritional program to support muscle maintenance and overall physical function in this population.

This study showed no effects on body composition, measured by appendicular lean soft tissue mass and muscle mass. A study with a similar exercise program in (pre)sarcopenic individuals found similar results [46], while most studies are not in line with our findings [47,48]. One possible explanation for the lack of effect is that the intensity of the resistance exercise training might be too low to elicit significant gains in muscle mass. Another explanation could be low commitment to the prescribed home-based exercises, resulting in participants failing to meet the recommended frequency and intensity of training. Indeed, the effectiveness of unsupervised home-based exercise interventions often remains inconsistent due to challenges such as adherence, motivation, and environmental factors [49]. Although we sought to address these barriers and establish a routine for home-based exercise, achieving consistent participation at home proved difficult and needs more guidance from a professional.

This lifestyle intervention combined group and individual sessions to incorporate different behaviour change techniques and enhances participant motivation [50,51]. Group-based exercise, supervised by a trainer, is widely recognized for its physical and social benefits, as well as enhancing intrinsic motivation [52,53]. The three physical therapist consultations had an adherence rate of 93%. However, in line with previous studies, no changes were observed in habitual physical activity levels, as assessed by accelerometers, suggesting the need for further exploration of strategies to enhance daily physical activity [54,55]. Similarly, adherence to the four dietitian consultations was high at 91%, leading to an increased protein intake by 24%, reaching 78 g/day. These findings suggest that ongoing professional guidance, including at least two personalized consultations over three months, may be beneficial in supporting improvements in protein intake [56]. Moreover, they underscore the necessity of a tailored, individualized approach rather than a one-size-fits-all strategy [57,58]. While the precise mechanisms underlying the observed improvements in physical performance and muscle strength maintenance remain unclear, our findings support the efficacy of a comprehensive approach combining an exercise program with sufficient protein intake to achieve long-term health benefits [49].

This culture-sensitive lifestyle intervention was specifically designed to address the socio-cultural needs of non-Western migrant older adults by integrating key culturally tailored elements. The intervention included group-based training, walking as preferred physical activity, education on protein-rich foods and exercise for healthy aging and practical guidance on protein intake. These elements were supported by culturally adapted educational materials and trained healthcare professionals. Group-based exercise was prioritized due to its strong social component which enhances motivation, adherence and long-term engagement in physical activity [52,53]. Walking was incorporated for its feasibility, cultural relevance, and acceptability among older migrant populations [59,60]. To address low nutritional literacy, the intervention provided culturally tailored education to improve knowledge, self-efficacy, and adherence to protein intake recommendations, which has been shown to support long-term dietary improvements [13,22]. Given the specific health challenges faced by this population, a culture-sensitive approach is critical for facilitating meaningful behavior change [[16], [17], [18], [19], [20],^60^]. By designing an intervention that considers the specific cultural values, dietary habits, and social dynamics, this study demonstrated the potential of culture-sensitive interventions to combat age-related conditions such as sarcopenia in non-Western migrant older adults.

This study had some strengths and limitations. A potential limitation may be the usage of bioelectrical impedance analysis to assess muscle mass, which may also explain our lack of a significant difference in muscle mass. While it has limited sensitivity and may not accurately detect small changes, it is a practical, accessible and non-invasive method that is well-suited for effectiveness studies [33]. The intended increase in physical activity could not be reliably measured due to low adherence to wearing ankle-mounted activity monitors. While some participants provided 3–5 days of complete data, others contributed only a single day, limiting the reliability of the assessment. This study included mostly females (91%), which limits the generalizability of the findings. While the intervention was effective for females, Surinamese older men are at higher risk for sarcopenia, greater effects may be observed in this population. Despite these limitations, key strengths of this study include its focus on a culture-sensitive intervention targeting an underrepresented population, and its approach to measuring all aspects of sarcopenia. By integrating culturally relevant elements such as group-based exercise, walking, and personalized dietary education, the intervention demonstrated the potential to improve outcomes related to sarcopenia in this population. This study could guide future research on culturally tailored interventions and their relevance for other non-Western migrant groups. Further studies with larger and more balanced cohorts are needed to build on these findings.

In conclusion, this study in non-Western Surinamese older adults revealed that a culture-sensitive lifestyle intervention improved physical performance, protein intake, and maintained muscle strength. This culture-sensitive intervention presents a promising approach for tackling sarcopenia in this vulnerable population.

## CRediT authorship contribution statement

PW, MT, JH and HK were involved in design of the project. JH, NS and MH were responsible for data collection and coordination of the trial. EB performed all the analysis. EB, JH, PW and MT drafted the manuscript. All authors approved the final manuscript.

## Ethics approval and consent to participate

This study was approved by the Medical Ethics Committee (METC) of the Amsterdam University Medical Centers, location VUmc, The Netherlands (NL75885.029.21). Written informed consent was collected from all participants.

## Consent for publication

Participants gave written informed consent to publish their data.

## Declaration of Generative AI and AI-assisted technologies in the writing process

The authors declare that they have not used any AI at all.

## Funding

This study is funded by the Taskforce for Applied Research SIA, part of the Dutch Research Council (NWO), grant number RAAK.MKB12.033.

## Data availability

The statistical analysis plan and data of this study is available in a data package on the Figshare repository with restricted access in consultation with open science principles (https://doi.org/10.21943/auas.28016189).

## Declaration of competing interests

The authors declare that they have no known competing financial interests or personal relationships that could have appeared to influence the work reported in this paper.
