# Supplementary material for: Culture-sensitive lifestyle intervention tailored to non-Western migrant older adults improves physical performance: A randomized controlled trial
Source: J Nutr Health Aging. 2025 May 22;29(8):100584. doi: 10.1016/j.jnha.2025.100584 (PMC12172971; doi:10.1016/j.jnha.2025.100584)
Supplement: Supplementary file 2 [file mmc2.docx]

**Appendix B. Development of the culture-sensitive lifestyle intervention “ProMIO”**

**Introduction**
The culture-sensitive intervention ProMIO has been developed through two sequential projects. The first project (2018-2020) employed qualitative methods with end-users and stakeholders, including Intervention Mapping methodology. A pilot study and process evaluation were conducted to assess feasibility and gather insights for refinement. In the second project (2021-2023), the intervention was improved and evaluated.

This manuscript presents the effectiveness of this newly developed intervention. This additional file 1 provides a comprehensive overview of the development and design of this culturally-sensitive, combined lifestyle intervention tailored for community-dwelling, non-Western migrant older adults.

**Project 1: Intervention mapping resulting in a intervention pilot**

Intervention Mapping (IM) is a structured protocol for designing theory-based and evidence-based health promotion programs (1). Focus groups and co-creating sessions with end-users and stakeholders are used as input for the intervention mapping.

Appendix 1 presents an overview of step 4 of the IM method, the developed components are defined including the underlying methods and behavioral determinants. This developed intervention was tested in the pilot of the first project.

*Key elements of the first intervention:*

- Biweekly progressive resistance exercise in a controlled gym setting with a physiotherapist (group training)
- Individual consultations with the physiotherapist and dietician
- Educational group sessions
- Duration: 12-week program
- Culture-sensitive approach for Turkish, Moroccan and Surinamese migrant older adults; including materials, training for health care professionals (HCPs).

## **Project 2: Suggested modifications and improvements for the intervention**

The major insights and results from the process evaluation of the first project are systematically presented here, highlighting challenges to address for adapting the new intervention and identifying positive aspects to retain.

**Key challenges:**

1. Challenges in the implementation of the manual:
   - Educational materials were incorrect and unclear
   - Health issues among participants necessitated stricter inclusion and exclusion criteria.
   - Participant motivation was inconsistent, highlighting the preference for healthcare professional (HCP)-led inclusion criteria.
   - Advice provided by dietitians lacked consistency.
2. Challenges in the intervention design:
   - Consultation and training durations were too short.
   - Screening criteria for participants required refinement.
3. Challenges in the organization:
   - Issues in communication.
   - Instances of inadequate collaboration.
   - Inefficient distribution of educational materials.

**Positive aspects:**

1. Positive points in the execution of the manual:
   - High motivation of adhering participants.
   - Effective use of body composition scales measuring muscle mass and body fat percentage.
2. Positive points in the intervention design:
   - Adequate number of consultations and training sessions.
   - Group training preferred exercise.
   - Educational materials tailored to participants' native language and culture.

## **Project 2: Adapted ProMIO intervention as described in this manuscript**

The enhanced ProMIO intervention, specifically designed for Turkish, Moroccan, and Surinamese migrant older adults, incorporates a culture-sensitive approach, including tailored materials and training for healthcare professionals. The program spans 6 months and emphasizes behavior change and real-world applicability.

Core components:

- Biweekly group-based exercise training: Conducted in a controlled setting with a physiotherapist or trainer, with session frequency decreasing over time.
- Home-based exercise program: Complements group training and involves unsupervised sessions, with frequency increasing over time.
- Physical activity: Walking is recommended as the preferred physical activity to increase aerobic capacity.
- Individual consultations: Sessions with a physiotherapist and dietician to provide personalized guidance.
- Educational group sessions: Focus on raising knowledge and awareness about protein-rich foods, exercise, and their role in healthy aging.
- Practical guidance: Support for integrating adequate protein intake into daily routines, while considering social norms, cultural values, and food preferences.

These core components were adapted to the social and cultural needs of the participants. Most relevant aspects related to the culture-sensitive intervention were:

**Theme:** **Social and cultural considerations**

- *Cultural similarity and mobilizing social support*: The group setting for both exercise and educational purposes fosters a supportive social environment. Since the group shares a common cultural background, participants are more likely to relate to each other, facilitating the professional's ability to address cultural aspects within their approach.
- *Individual consulting and tailoring*: Individual consultations are essential for personalizing the intervention. They also allow for the integration of techniques such as goal-setting, motivational interviewing, set graded tasks. As well enhance the self-efficacy and skills of the older adults by providing personal feedback on behavior and progress. These consultations are particularly important for addressing individual challenges in behavior change and ensuring the sustainability of behavior when contact with professionals decreases over time.
- *Role modeling*: From a cultural perspective, role models are vital for establishing relatedness. However, healthcare professionals (HCPs) typically lack diverse cultural and ethnic backgrounds. Consequently, additional training is required for HCPs, as they do not naturally serve as role models for this population. Training for HCPs was implemented to bridge gaps in cultural competence.

**Theme: Behaviour change strategies**

- *Guided practice vs self-practice*: Guided practice (in a group) has the preference for this population. However, the cost of maintaining guided practice (at least twice a week over a 6-month period) presents a challenge. As a result, professional support was gradually reduced over time. The intervention aimed to transition participants from group exercise to self-practice in the home environment. In educational sessions and individual consultations, professionals placed additional emphasis on habit formation, (small) goal setting and coping strategies.
- *Providing cues for motivation:* WhatsApp reminders, visible cues like the protein posters, and pocket folders in participants’ homes supported adherence to behavior change.
- *Low literacy adaptations:* To enhance the knowledge and self-efficacy determinants of behavior, the materials provided were designed to be visually appealing, simple, with minimal text, and culturally sensitive to the target group’s specific needs.

**Theme: Practical implementation**

- *Real-world setting*: The intervention is designed to be delivered by a regular physiotherapist, trainer and dietician. The exercises in the group sessions have similarity with the exercises at home to increase the self-efficacy. The aimed protein intake increase is encountered by regular habitual food intake, with consumer foods from their supermarkets. If needed, personalization of food intake could include budget considerations.
- *Training for Professionals*: In addition to protocol and logistics training, HCPs participated in culturally sensitive workshops with knowledge provided by the Pharos Knowledge Center, focusing on health equity and low-literacy strategies. Monthly peer consultations enhanced interprofessional collaboration.

Regarding the core components of the intervention and the social and cultural needs of the participants, the following intervention aspects were adapted and developed using the Intervention Mapping methodology:

- - Training for professionals: Physical and e-learning modules.
  - Peer consultation for professionals: Monthly interprofessional meetings.
  - Professional resources: Intervention manuals and online participant data folders. (Communication and storage of data).
  - Multicomponent exercise manuals: For both group and home-based exercises.
  - Participant materials: Intervention map for planning of visits, manuals, and access to an online library of exercise videos.
  - Resistance training equipment: Simple, low cost materials as free weights and band.
  - Group sessions: Scheduled sessions focused on exercise (weeks 5, 12), protein education (weeks 3, 7, 15), and sustainability after the program (week 25).
  - Educational tools: Protein posters, pocket folder, and culturally adapted protein variation food list.
  - Intervention material: Protein posters were updated with protein blocks and grams per portion. To maintain its simplicity, an additional protein pocket folder was created, featuring general information, a protein variation list, and culturally specific recipes. These materials were distributed before the intervention started to participants and professionals.
  - Online instruction video’s: QR codes linking to an online instruction video about protein and exercises were integrated.

**Structure of the core components of the intervention**

The following Figure 1 presents the core components of the ProMIO lifestyle intervention in the six-month timeframe.

**
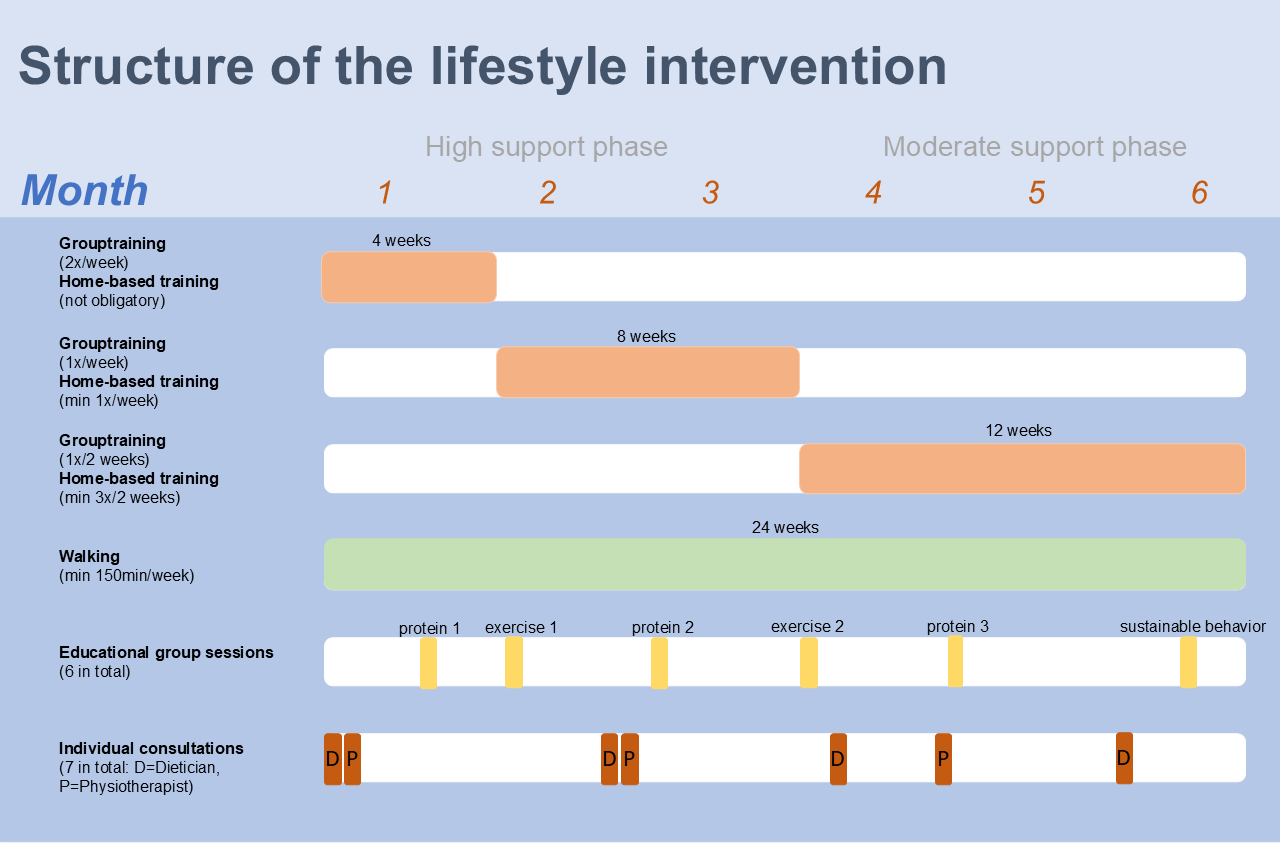

Figure 1. Core components of the 6 month ProMIO lifestyle intervention**

**Summary and website**

This document summarizes the iterative development and cultural adaptations of the culture-sensitive ProMIO intervention to enhance its feasibility, accessibility, and effectiveness for older adults in diverse community settings.

The role of the professional and the role of the behavior change aspects are crucial in this type of combined lifestyle intervention. The process evaluation of ProMIO will gain more insights in the current lessons learned, challenges and opinions of the stakeholders.

We have a project website available containing this development, intervention materials and future output and results: [Promio - Voeding & Beweging.NU](https://voedingenbeweging.nu/promio/)

**References**
1. Bartholomew Eldridge, L. K., Markham, C. M., Ruiter, R. A. C., Fernàndez, M. E., Kok, G., & Parcel, G. S., 2016. *Planning health promotion programs; an Intervention Mapping approach*, 4th Ed. San Francisco, CA: Jossey-Bass. [ISBN](https://en.wikipedia.org/wiki/ISBN_(identifier)) [978-1119035497](https://en.wikipedia.org/wiki/Special:BookSources/978-1119035497)

| **Appendix 1: Comprehensive overview of step 4 of Intervention Mapping** | | | |
| --- | --- | --- | --- |
| **Tabel S1. Step 4 of IM: overview of components, related change objectives, considered methodologies and related behavioral determinants.** | | | |
| **What? (component)** | **Complementing Change objectives** | **Methodologies** | **Behavioral determinants** |
| **Information sessions and workshops in the native language:**  1) Introduction to ProMIO; what to expect  2) Importance of proteins and strength training  3) The social environment; how to say no?  4) Proteins: where to find them?  5) Strength training: how do I fit this into my daily life?  6) Proteins: how can I cook with them? | 2. A.1. OE.1. OE.2. OE.1. OE.2. OE.4. OE.5. AT.1. AT.1.  3. PN.1.1 PN.1.2. 4. AT.4. PN.3.1. K.3.  5. PN.1.1. S.4. K.3.  6. AT.4. PN.4.1. S.4.1. S.4.2. K.4. | **Individual:** Providing cues, Modeling, public commitment (3), Active learning, Using imagery, planning coping responses (3), Arguments, Cultural similarity, resistance to social pressure (3), Mobilizing social support, Guided practice **Environment:** Environmental reevaluation, shifting perspective | **Individual:** Habits, Knowledge, Skills, Attitude, Outcome expectations, Social influence **Environment:** Awareness, Attitude, Outcome expectations, Self-efficacy |
| **Biofeedback** | A.4. A.4. SE.4. SE.5. | Self-monitoring of the behavior, personalize risk | Awareness, self-efficacy |
| **Intake with physiotherapist and dietician** | PN.2. | Verbal persuasion | Self-efficacy, skills |
| **Consultations with the dietician** | SE.1. SE.4. SE.5. OE.5. AT.1. PN.2. S.2. K.2. | Tailoring, self-reevaluation, verbal persuasion, goal setting, set graded tasks, motivational interviewing | Attitude, outcome expectations, self-efficacy, skills |
| **Consultations with the physiotherapist** | SE.1. SE.3. SE.4. OE.4. AT.1. PN.2. S.2. | Tailoring, self-reevaluation, verbal persuasion, goal setting, set graded tasks, motivational interviewing | Attitude, outcome expectations, self-efficacy, skills |
| **Twice a week progressive strength training** | S.3. SE.3. | Tailoring, guided practice, set graded tasks | Skills, self-efficacy |
| **Group app contact (sports)** | SE.1. SE.4. PN.3.2 | Public commitment | Habits, Self-efficacy |
| **Reminders via WhatsApp** | M.2. M.2. | *Providing cues* | Self-efficacy, motivation |
| **DIY materials for home:** 1) Videos for reference (vlogs, tutorials, and educational content, whiteboard markers)  2) Poster with protein-rich products  3) Poster with strength exercises | K.1. K.1. K.3. K.4. K.3. K.4. S.4.1. S.4.2. S.3. S.4. OE.5. OE.4. | Providing cues, using imagery, Modeling, Cultural similarity | Habits, Knowledgde, Skills, Attitude |
| **Two training sessions for health care professionals:** 1) Cultural aspects  2) Engaging with the target group | A.3. A.4. SE.1.1. SE.1.2. SE.3. S.2.1. S.2.2. S.3.1. S.4.1. K.2.1. K.2.2. K.3.1. K.3.2. K.4. | Providing cues, guided practice, enactive mastery experiences, self-monitoring of behavior | Knowledge, Self-efficacy Skills, Awareness |

**Appendix 2: Example materials (poster and pocket booklet)**

**Figure 1. Protein poster**


The design of the poster was updated with protein blocks and grams per portion were introduced. To maintain its simplicity, an additional protein pocket folder was created, featuring general information, a protein variation list, and culturally specific recipes. Figure 3 shows a screenshot of the protein poster, while Figure 4 highlights a page from the pocket folder that emphasizes portion sizes with a recipe.


 **Figure 2. Protein pocket booklet**
