# Supplementary material for: Culture-sensitive lifestyle intervention tailored to non-Western migrant older adults improves physical performance: A randomized controlled trial
Source: J Nutr Health Aging. 2025 May 22;29(8):100584. doi: 10.1016/j.jnha.2025.100584 (PMC12172971; doi:10.1016/j.jnha.2025.100584)
Supplement: Supplementary file 3 [file mmc3.docx]

# Appendix C: Per protocol analysis

Table 4: Per protocol (≥80% adherence to group training sessions) effects of the lifestyle intervention on physical performance and muscle mass outcomes

|  |  |  |  |  |  | Linear mixed models with interaction effects ^*^ | | Adjusted linear mixed models with interaction effects ^ƚ^ | |
| --- | --- | --- | --- | --- | --- | --- | --- | --- | --- |
|  |  | Intervention group | | Control group | | Intervention effects |  | Intervention effects |  |
| Outcome variable |  | n | Mean ± SD | n | Mean ± SD | Difference (95% CI) | P-value | Difference (95% CI) | P-value |
| 6-minute walk test (m)  Timed up and go (s)  30-seconds chair stand test  Knee-extension strength (N)  Open eyes single leg stand (s)^a^  Closed eyes single leg stand (s)^a^  Body weight (kg)  Appendicular lean mass (kg)^a^  Fat mass (kg)  Cross-sectional area Rectus Femoris (cm^2^)  Cross-sectional area Vastus Lateralis (cm^2^) | 0 months  3 months  6 months  0 months  3 months  6 months 0 months  3 months  6 months 0 months  3 months  6 months  0 months  3 months  6 months  0 months  3 months  6 months  0 months  3 months  6 months 0 months 3 months 6 months 0 months  3 months  6 months 0 months  3 months  6 months  0 months  3 months  6 months | 19  19  18  19  19  19  19  19  1919  19  19  19  19  19  19  19  19  19  19  19  18  19  19  18  19  19  19  19  19  18  19  19 | 449 ± 63  481 ± 71  493 ± 69  7.9 ± 1.8  7.5 ± 1.5  7.4 ± 1.2  10 ± 3  11 ± 3  11 ± 2  273 ± 78  268 ± 68  262 ± 73  26 (15-42)  16 (7-37)  39 (11-47)  3.5 (2.6-5.3)  1.8 (1.5-2.2)  3.2 (1.8-4.3)  76.0 ± 13.6  75.5 ± 13.2  75.7 ± 13.2  17.4 ± 2.9  17.3 ± 2.8  17.4 ± 2.8  32.6 ± 8.9  32.3 ± 8.2  32.2 ± 8.1  4.3 ± 1.1  4.1 ± 1.0  4.5 ± 1.0  16.0 ± 1.7  15.8 ± 3.2  15.8 ± 2.9 | 30  27  24  30  28  24  30  28  24  30  28  24  30  28  24  30  28  24  30  28  2430  28  24  30  28  24  30  26  24  29  25  24 | 438 ± 93  455 ± 87  471 ± 66  8.0 ± 1.8  7.3 ± 1.3  7.3 ± 1.4  11 ± 3  11 ± 2  11 ± 2  262 ± 78  263 ± 82  240 ± 87  31 (15-78)  26 (10-77)  35 (10-101)  3.6 (2.3-5.6)  2.9 (1.6-5.4)  3.6 (1.8-6.1)  77.2 ± 16.4  75.6 ± 16.7  77.2 ± 18.1  16.9 ± 3.5  16.6 ± 3.6  17.2 ± 4.0  35.1 ± 11.0  34.5 ± 11.1  34.5 ± 12.1  4.9 ± 1.6  4.5 ± 2.1  4.9 ± 2.2  15.6 ± 5.0  15.7 ± 4.9  15.8 ± 5.7 | +17.5 (-7.4; 42.4)  +29.5 (3.8; 55.3)  +0.3 (-0.2; 0.9)  +0.2 (-0.4; 0.8)  +0.3 (-1.1; 1.7)  +0.1 (-1.3; 1.6)  -2 (-22; 19)  +17 (-5; 38)  -3 (-22; 16)  -3 (-25; 14)  -3 (-7; 1)  -3 (-7; 1)  -0.1 (-1.4; 1.3)  -0.7 (-2.1; 0.7)  +0.2 (-0.2; 0.5)  -0.1 (-0.5; 0.3)  -0.6 (-1.5; 0.3)  -0.6 (-1.6; 0.4)  -0.1 (-0.7; 0.6)  -0.1 (-0.7; 0.6)  -0.1 (-2.0; 1.9)  +0.1 (-1.8; 2.1) | 0.171  **0.027**  0.247  0.479  0.663  0.855  0.886  0.133  0.788  0.577  0.072  0.100  0.910  0.307  0.425  0.555  0.214  0.223  0.795  0.940  0.975  0.897 | +20.1 (-4.6; 44.9)  +32.9 (6.4; 57.5)  +0.3 (-0.3; 0.8)  +0.2 (-0.4; 0.7)  +0.4 (-1.0; 1.8)  +0.2 (-1.2; 1.7)  -1 (-22; 20)  +17 (-4; 39)  -4 (-22; 15)  -6 (-26; 13)  -3 (-7; 1)  -3 (-7; 1)  +0.1 (-1.4; 1.4)  -0.7 (-2.1; 0.8)  +0.2 (-0.2; 0.6)  -0.1 (-0.5; 0.3)  -0.6 (-1.6; 0.3)  -0.6 (-1.6; 0.3)  -0.1 (-0.7; 0.6)  -0.1 (-0.7; 0.7)  +0.2 (-1.7; 2.1)  0.3 (-1.6; 2.3) | 0.114  **0.016**  0.331  0.606  0.572  0.759  0.919  0.124  0.700  0.521  0.065  0.096  0.984  0.364  0.342  0.653  0.176  0.181  0.854  0.988  0.856  0.740 |

Data are presented as mean ± SD for each time point. Data were analysed using Linear Mixed Models with time and time x intervention interaction as fixed effects and subjects were added as a random intercept. Control group and baseline values are used as reference. ^a^ Appendicular lean mass was assessed by BIA and estimated using the formula of Sergio et al. (2016).
^*^ Crude β-coefficients and 95% CIs are shown for the time x intervention interaction.
^ƚ^ Adjusted β-coefficients and 95% CIs are shown for the time x intervention interaction. Adjusted for age, sex and BMI.
^a^ Data for this outcome is presented as median with interquartile range.

Table 5: Per protocol (≥80% adherence to group training sessions) effects of the lifestyle intervention on behavioural outcomes

|  |  |  |  |  |  | Linear mixed models with interaction effects ^*^ | | Adjusted linear mixed models with interaction effects ^ƚ^ | |
| --- | --- | --- | --- | --- | --- | --- | --- | --- | --- |
|  |  | Intervention group | | Control group | | Intervention effects |  | Intervention effects |  |
| Outcome variable |  | n | Mean ± SD | n | Mean ± SD | Difference (95% CI) | P-value | Difference (95% CI) | P-value |
| Protein intake (g/day)  Protein intake (g/adjusted kg/day)  Light physical activity (minutes)  Moderate physical activity (minutes)  Heavy physical activity (minutes)  Total physical activity (minutes) | 0 months  3 months  6 months 0 months  3 months  6 months 0 months  3 months  6 months 0 months  3 months  6 months  0 months  3 months  6 months  0 months  3 months  6 months | 19  19  19  19  19  19  16  17  16  16  17  16  15  17  16  16  17  16 | 61 ± 15  94 ± 34  90 ± 39  0.9 ± 0.2  1.4 ± 0.6  1.3 ± 0.5  121 ± 54  110 ± 34  107 ± 51  69 ± 30  58 ± 23  67 ± 26  5 ± 5  5 ± 7  4 ± 3  196 ± 79  172 ± 49  178 ± 72 | 30  28  24  30  28  24  25  26  21  25  26  2123  26  21  25  26  21 | 72 ± 25  71 ± 23  78 ±29   - 1. ± 0.4   1.1 ± 0.3  1.2 ± 0.5  94 ± 38  87 ± 24  101 ± 35  54 ± 27  54 ± 27  54 ± 21  5 ± 5  4 ± 4  4 ± 3  153 ± 62  145 ± 51  159 ± 53 | +26 (10; 41)  +15 (-1; 31)  +0.4 (0.1; 0.6)  +0.2 (-0.1; 0.4)  +3 (-15; 21)  -4 (-24; 15)  -1 (-16; 15)  +9 (-7; 25)  +1 (-2; 3)  -1 (-3; 3)  +2 (-30; 34)  +4 (-31; 38) | **0.002**  0.069  **0.003**  0.138  0.773  0.671  0.943  0.269  0.687  0.976  0.895  0.841 | +25 (10; 41)  +15 (-2; 31)  +0.4 (0.1; 0.6)  +0.2 (-0.1; 0.4)  +3 (-16; 21)  -4 (-24; 15)  +1 (-15; 15)  +10 (-6; 25)  +1 (-2; 3)  +1 (-2; 3)  +4 (-28; 36)  +4 (-30; 38) | **0.002**  0.080  **0.003**  0.167  0.771  0.669  0.962  0.240  0.472  0.896  0.818  0.805 |

Protein intake was corrected for BMI > 27.5.
Data are presented as mean ± SD for each time point. Data were analysed using Linear Mixed Models with time and time x intervention interaction as fixed effects and subjects were added as a random intercept. Control group and baseline values are used as reference.
^*^ Crude β-coefficients and 95% CIs are shown for the time x intervention interaction.
^ƚ^ Adjusted β-coefficients and 95% CIs are shown for the time x intervention interaction. Adjusted for age, sex and BMI. Protein intake (g/adjusted kg/day) was adjusted for age and sex.
